# Therapeutic duplication of anticoagulants: a retrospective study of frequency and consequences in a tertiary referral hospital

**DOI:** 10.1186/s12959-020-00227-w

**Published:** 2020-08-03

**Authors:** Ramin Rahmanzade, Francisco Cabrera Diaz, Claudia Zaugg, Philipp Schuetz, Ali Reza Salili

**Affiliations:** 1 Faculty of Medicine, University of Basel, Basel, Switzerland; 2 Biomedical Research and Training, University Hospital Basel, Basel, Switzerland; 3 Hospital Pharmacy, Kantonsspital Aarau, Aarau, Switzerland; 4 Department of Internal Medicine, Kantonsspital Aarau, 5000 Aarau, Switzerland; 5 Department of Internal Medicine, Clinical Pharmacology, Kantonsspital Aarau, Tellstrasse 51, CH-5001 Aarau, Switzerland

**Keywords:** Anticoagulants, Therapeutic duplication, Medication error, Direct Oral anticoagulants, HAS-BLED score

## Abstract

**Background:**

Anticoagulants are commonly prescribed in medical practices and could be of significant harm in the case of medication errors. We conducted a retrospective observational study to determine the frequency and consequences of the therapeutic duplication of anticoagulants (TDA). As a secondary objective, we aimed to determine the characteristics of the population in which TDA occurs.

**Methods:**

We conducted a retrospective observational study among admitted patients who concomitantly received at least two anticoagulants from August 2017 to August 2018.

**Results:**

A total of 107 patients with TDA are included in the research. The patients with TDA have a mean age of 73. The TDA population has a high rate of associated comorbidities with 69% of patients having arterial hypertonia, 40% with chronic kidney disease, 26% with a history of malignancy, and 20.5% with a history of stroke. More than 65% of patients were under anticoagulation before admission, mostly due to atrial fibrillation. The TDA occurred in more than 95% of cases in the first week or the last week of hospitalization. Patients had a high risk of bleeding prior to the TDA-event with about 62.5% of TDA patients having a HAS-BLED score at least 3. A total of 8 patients showed a significant Hemoglobin (Hb)-drop of at least 10 g/L within 24 h after TDA-event. Two patients had a new or worsened hematuria following TDA-event.

**Conclusion:**

TDA occurred in 0.8% of patients who were under anticoagulation and in 6.7% of patients who received direct oral anticoagulants (DOACs). TDA led in about 7.4% of cases to hemoglobin-relevant bleeding. The old patients with significant comorbidities and a high HAS-BLED score were mainly affected. The female gender and presence of anemia independently predicted the occurrence of bleeding following TDA.

## Background

According to the Institute for Safe Medication Practice (ISMP), anticoagulants (AC) are among the high-alert-medications leading to serious complications in the case of medication errors [1]. Anticoagulants are involved in about 8.3% of all medication errors in admitted patients [2]. In a 5-year retrospective study, the investigators showed that medication errors are the cause by about 48.8% of all AC-associated adverse drug events [3]. The AC-related medication errors have been mainly classified according to the phase of medication process in which the error occurred into the following types: prescribing, transcribing, dispensing, administering, and monitoring errors. A cross-sectional study on the nation-wide drug safety system of the Netherland showed that the anticoagulant-related medication errors occurred mainly in the prescribing phase and in about 56% of errors low-molecular-weight heparins (LMWH) were involved [2, 4]. Consistently, a similar descriptive study on the Danish patient safety database reported that the adverse medication incidents occurred mainly in the prescribing phase, and in about 80% of incidents the errors occurred at the time of sector changes (at the admission, at the discharge or ward changes) [5]. Among different error subtypes occurring in the prescribing phase, the wrong dosage and drug omission have been repeatedly found as the most common subtype [3, 6, 7]. The affected patients are usually polymorbid patients in advanced ages having already high risk of bleeding [8]. Accordingly, the AC-related errors occur in about 78% of cases in adults older than 60 and in about one third in patients older than 80 years old [9]. The studies reporting the frequency and complications of AK-related medication errors have been mostly done based on data reported to in-house or nation-wide drug safety systems [2, 3, 5–10]. As many of these errors could not be detected or have not been reported by medical staff, the studies based on them could not reliably show the incidence of errors, the affected population and the rate of consequences. For different categories of ACs, the rates of adverse complications, especially the minor and major bleeding rates, have been well studied and reported [11–15].

Therapeutic duplication is defined a concomitant prescription of two medications of the same class. Except for a few indications, the therapeutic duplication of anticoagulants is contraindicated. The therapeutic duplication of anticoagulants (TDA) as a medication error occurring in the prescribing phase was rarely studied [15–17]. As the primary objective, we aimed to comprehensively investigate the incidence, affected population, and complications of TDA in patients admitted to our tertiary referral hospital from 21.08.2017 to 20.08.2018.

## Material & Methods

### Patient population

This study is a retrospective observational study conducted at Kantonsspital Aarau (KSA), a 450-bed tertiary care facility providing medical and surgical care in all general and specialized medical disciplines. The hospital utilizes an electronic health care system (KISIM) gathering all medical and personal data of patients who received medical care, either in the in-patient or out-patient settings. For the present study, we retrospectively collected data of admitted patients who received AC, using a computerized export from KISIM. From this population, all patients admitted to our in-patient wards, from 21.08.2017–20.08.2018, who concomitantly received at least two ACs were included in the study. Subsequently, the following patients have been excluded: [1] patients who received the anticoagulants separately, no overlapping prescription [2] patients treated with the combination of heparin/ heparin analogs and vitamin-K antagonists (VKA) [3] patients receiving heparin/ heparin analogs based on a weekly schedule such as patients with hemodialysis [4] patients receiving low-dose alteplase (= < 4 mg) as a catheter locking solution [5] patients treated with the combination of VKA and direct oral anticoagulants (DOACs) during bridging from DOACs to VKA [6] patients who explicitly refused participation in research.

### Data collection and data analysis

An export (in CSV format) from the data storage system of our hospital (KISIM) has been done. The mentioned export includes all patients who received at least one anticoagulant during their admission from 21.08.2017–20.08.2018 and contains the following patient data: Patient number, admission number, sex, date of birth, dates of admission and discharge, the name of prescribed anticoagulants and the dates of prescriptions.

According to the inclusion and exclusion criteria, the patients with a contraindicated combination of at least two AK have finally been separated. Then, the corresponding demographic, biochemical and clinical parameters, listed ahead were extracted from KISIM: The para-clinical data including pre-error and post-error Hb (Hemoglobin of patient before and after TDA), pre-error and post-error urine analysis, the indication of anticoagulant therapy, the presence of underlying diseases predisposing, the patients to develop complications under TDA: arterial hypertension, renal insufficiency and its stage according to glomerular filtration rate (GFR), hepatic failure, active gastrointestinal ulcers, concomitant therapy with NSAIDs or thrombocyte-aggregation inhibitors, documented bleeding diathesis (abnormal post-operative bleeding, hematoma or bleeding under anticoagulant therapy in therapeutic dosage, known hemorrhagic disorders, etc.), prior anticoagulant therapy, history of recent trauma or upcoming surgery and finally the HAS-BLED score prior to the error. Subsequently, all demographic, biochemical and, clinical data of patients included in the study have been analyzed statistically. The variables have consequently been analyzed descriptively. A Hemoglobin-drop of more than 10 g/L within 24 h has been considered as significant Hb-drop. The patients have been divided into two groups with and without significant Hb-drop. Then, unpaired T-test and chi-square have been done for continuous and binary variables, respectively, to study whether there is a significant difference in variables between two patients groups. Finally, to determine the effect of different risk factors on the bleeding following TDA, we performed univariate and multivariate logistic regression.

## Results

**Fig. 1:**
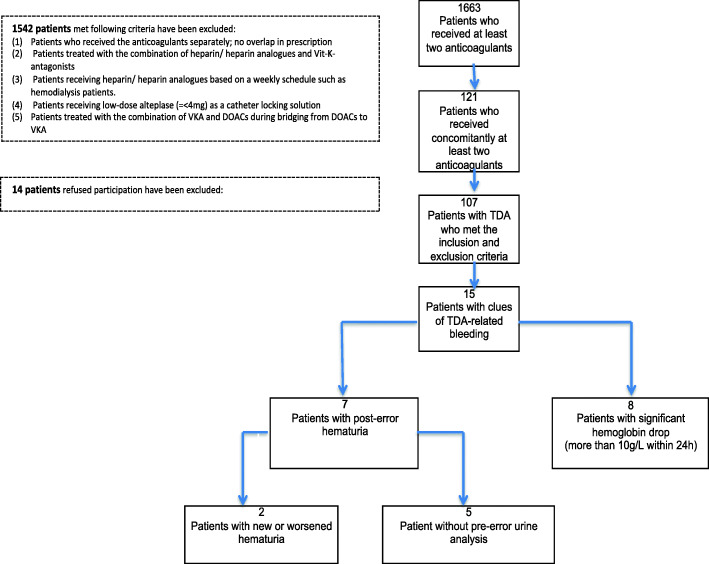
Diagram of patient recruitment

Among 14,812 patients who received at least one anticoagulant, a total of 1663 patients met the inclusion criteria and have been treated with at least two anticoagulants during the one-year period. Among them, a total of 1542 patients met the first five exclusion criteria. Then, a total of 121 patients with a contraindicated combinative therapy of at least two anticoagulants (TDA) have been found. Out of them, 14 patients refused to participate and the rest 107 patients were included in the study (Fig. 1).

### Characteristics of the patient population

#### Age and sex

From 107 patients included in the study, 72 patients (67.3%) were male and 35 patients (32.7%) were female. The minimum and maximum of age were 35 and 97 years, respectively. The mean age of patients was 73.67 (95% CI, 71.2–76.1) and 78.5% of the population was older than 65 years.

### Duration of hospitalization

The minimum and maximum of the hospitalization duration were 2 and 72, respectively. The mean duration of hospitalization was 11.52 (CI 95%: 9.69–13.3).

### HAS-BLED score related comorbidities

Table 1 shows the HAS-BLED score related parameters and comorbidities. Out of 107 patients, 74 patients (69.1%) had long-term arterial hypertension. Among them, 38 patients had uncontrolled arterial hypertension with systolic blood pressure higher than 160 mmHg. A high proportion of patients (40.1%) had a known chronic kidney disease prior to admission. Among them, 69.7% had stage 3 or higher. Abnormal liver function has been found in 7 patients; 2 with liver cirrhosis and 5 others with hepathopaties. 20.5% of patients had a history of stroke. A relevant co-medication with other drugs (aspirin, clopidogrel, NSAIDs etc.) or alcohol consumption predisposing patients to bleed has been found in 28 Out of 107 patients (26%). Seventeen patients were known to have a bleeding diathesis or a history of major bleeding (See Table 1).

**Table 1:** A summary of the HAS-BLED score, the modified score and the related parameters

Characteristics	TDA-patients (whole population)	Patients with Hb-relevant bleeding	Patients without Hb-relevant bleeding
Number	107	8	99
Hypertension	38	4	34
Abnormal liver function	7	1	6
Abnormal renal function	43	3	40
Stroke	22	3	19
Labile INR	15	2	13
Bleeding diathesis	17	2	15
Age > 65 years	84	7	77
Drug/alcohol concomitantly	28	2	26
Prior anticoagulation	71	7	64
HAS-BLED score	2.37 (CI95% 2.14–2.60)	3.00 (CI95% 2.26–3.74)	2.32 (CI95% 2.08–2.55)
Modified HAS-BLED score	3.04 (CI95% 2.8–3.28)	3.87 (CI95% 3.09–4.66)	2.97 (CI95% 2.72–3.22)

### HAS-BLED score of the patient population

The minimum and maximum values of the HAS-BLED scores were 0 and 7, respectively. The mean HAS-BLED score was 2.37 (CI95% 2.14–2.60) with a median value of 3.

### Further bleeding-related parameters and comorbidities

Table 2 shows further bleeding-related comorbidities, which have not been considered by the HAS-BLED score. Most of the patients with TDA (66.3%) were on anticoagulation prior to admission. Diabetes mellitus has been found in 24 patients (22.4%). A high proportion of patients had a history of malignancy (26.1%). Out of 107 included patients, 22 (20.5%) and 13 (12.1%) patients had ischemic and congestive heart disease, respectively. A fresh fracture or traumatic brain injury has been found in 12 patients (11.2%) at admission. A total of 13 patients had a documented thrombocytopenia prior to the medication error and 8 patients had a known falling tendency. The higher proportion of patients with falling tendency, ischemic heart disease or with a history of a recent fracture or brain injury in the group of patients with Hb-relevant bleeding may put this population at increased risk of complications following TDA. Although it was suggested that anemia might contribute to the major bleeding under rivaroxaban therapy [18], our study showed a lower proportion of anemia in patients with Hb-relevant bleeding. However, the risk of bleeding may also be affected by other contributing factors. It has been demonstrated that AC therapy in patients with deep vein thrombosis leads more frequently to bleeding complications compared to those with atrial fibrillation [19, 20]. While 50% of patients with Hb-relevant bleeding were under anticoagulation due to AF, this was 68% for patients without Hb-relevant bleeding.

**Table 2:** Comparison of bleeding-relevant characteristics of TDA-patients with and without significant Hb-drop

Parameters	TDA-patients (whole population)	Patients with Hb-relevant bleeding	Patients without Hb-relevant bleeding
Pre-error anemia	63.5%	37.5%	65.6%
Pre-error thrombocytopenia	12.1%	12.5%	12.1%
History of malignancy	26.1%	0%	26.1%
Diabetes mellitus	22.4%	0%	22.4%
Ischemic/ congestive heart disease	IHD 20.5% CHF 12.1%	IHD 37.5% CHF 12.5%	IHD 19.1% CHF 12.1%
Recent fracture or traumatic brain injury	11.2%	37.5%	0.9%
Falling tendency	7.5%	12.5%	0.7%
Death in 2 years	18.6%	25%	18.1%

### Modified HAS-BLED score

Prior anticoagulation is not considered in the HAS-BLED score as a parameter. Indeed, the HAS-BLED score is employed to assess the risk of bleeding following initiation of an anticoagulative therapy in patients who were not being anticoagulated before. As taking anticoagulants before admission might have predictive value for hemorrhagic complications, we defined and applied a modified score by considering the prior anticoagulation as a criterion as well. Another rationale for employing the modified value is the above-mentioned finding that more than 66% of TDA patients were on anticoagulation therapy before admission. The modified HAS-BLED score was calculated by adding one score for prior anticoagulation to the HAS-BLED score. Using the modified HAS-BLED score, the TDA population had a mean value of 3.04 (CI95% 2.8–3.28) and about 67.2% of TDA patients had a score of at least 3.

### Rate of anemia prior to medication error of TDA

For 8 patients, no hemoglobin value has been found within 72 h prior to TDA. The rest population with available pre-error hemoglobin had a mean hemoglobin value of 120.7 (95%CI: 116–125). 47 out of 72 male patients (65.2%) had a hemoglobin value lower than 140 g/L prior to error. From 35 females with TDA, 21 patients (60%) had a hemoglobin value lower than 120 g/L prior to error. Altogether, 63.5% of TDA patients had anemia prior to medication error.

### TDA-related parameters

#### Duration of TDA

TDA lasted, before detection and stop, for a minimum and maximum of 1 and 7 days, respectively. The mean duration of TDA was 1.68 (95%CI: 1.42–1.94).

#### Time interval of the error to admission or discharge

TDA events began in a short interval to the admission or discharge with a mean interval of 2.15 (95%CI: 1.67–2.63). In 21.4% of patients the TDA began on the day of admission or discharge. In 86 patients (83.3%) the error began in within 72 h after admission or before discharge (Table 3).

**Table 3:** Comparison of characteristics of TDA between the whole population and the patients with Hb-relevant bleeding

Parameters	TDA-patients (whole population)	Patients with Hb-relevant bleeding
Duration of TDA	1.68 (SD 1.35)	1.75 (SD 1.38)
Time interval to admission or discharge	2.15 (SD 2.55)	1.125 (SD 0.99)
Ward	Medical 38.3% Surgical 61.6%	Medical 62.5% Surgical 37.5%
Setting	27.1% post-op 8.4% pre-op	25% post-op 12.5% pre-op
Involved anticoagulants	LMWH+DOAC (88.7%) Heparin+DOAC (7.5%) LMWH+Heparin (0.9%) DOAC+DOAC (0.9%) VKA + Heparin+LMWH (0.9%)	LMWH+DOAC (87.5%) LMWH+Heparin (12.5%)

#### Involved anticoagulants

Most of TDA patients (88.7%) had a combination of LMWH and DOAC. Among them, 43 patients (40.1%) have been treated with the combination of rivaroxaban and dalteparin and 40 patients (37.3%) with the combination of apixaban and dalteparin as the most common combination of anticoagulants (Table 4).

**Table 4:** The anticoagulants involved in the TDA events of 107 patients

Involved ACs	Nr. of patients	Proportion
Rivaroxaban+Dalteparin	43	40.1%
Apixaban+ Dalteparin	40	37.3%
Dabigataran+ Dalteparin	7	6.5%
Edoxaban+ Dalteparin	5	4.6%
Liquemin+ Rivaroxaban	3	2.8%
Liquemin+Apixaban	2	1.8%
Liquemin+ Dalteparin	2	1.8%
Edoxaban+Heparin	1	0.9%
Liquemin+Dabigatran	1	0.9%
Apixaban+rivaroxaban	1	0.9%
apixaban+Arixtra	1	0.9%
Marcoumar+Liquemin+Dalteparin	1	0.9%

In total, 1737 patients received at least one DOAC during the study period. Among them, 104 patients who were included in the study (See Table 4) and all of the excluded TDA patients i.e. fourteen patients were under DOAC at the time of TDA. Thus, TDA occurred in 6.7% of patients who were on DOAC.

#### Significant Hb-drop following TDA

To evaluate the bleeding events following TDA, we considered the last hemoglobin value reported within 72 h prior to TDA and the fist hemoglobin value reported within 72 h after TDA. A significant Hb-drop, which could be attributed to the bleeding, has been defined as a drop of more than 10 g/L within 24 h. The patients with Hb-drops, which could be attributed to other etiologies rather than TDA such as infusion therapies, operations, or known bleeding sources prior to TDA have been excluded. A total of 8 patients showed significant Hb-drop, which could not be attributed to the above-mentioned etiologies; please see the diagram of patients recruitment. Among patients with significant Hb-drop, two patients were taking platelet-aggregation inhibitors before admission; one was on acetylsalicylic acid 100 mg daily and the other one was on clopidogrel 75 mg daily (See case 2 in the part *case study*). Both drugs were stopped upon admission i.e. none of the patients with significant Hb-drop was receiving any platelet-aggregation inhibitors at the time of TDA events.

#### Bleeding risk factors in TDA patients

Unpaired T-test and Chi-square have been done for all continuous and binary variables, respectively, to study whether there is a significant difference of variables between two patients groups; with and without Hb-drop. A *p*-value under 0.05 was defined as statistically significant. The relative risks for binary variables are also reported in Table 4. The modified HAS-BLED score was the only variable that reached the statistical significance in the univariate analysis (*p* = 0.028). HAS-BLED score did not reach the significance (*p* = 0.064). Although the TDA patients with a history of prior anticoagulation may have been at increased risk of bleeding (RR 3.55, CI95% 0.45–27.75), this risk was not translated into statistically significant TDA events (*p* = 0.22) (Table 5).

**Table 5:** Univariate Analysis results

Risk factor	Odds ratio	Relative risk
Anemia	0.17 (CI 95% 0.03–0.87)	0.19
Abnormal renal function	0.78 (CI 95% 0.18–3.45)	0.8
Uncontrolled hypertention	1.91(CI 95% 0.45–8.12)	1.82
Stroke	2.53 (CI 95% 0.55–11.51)	2.32
Age > 65	2.0 (CI 95% 0.23–17.14)	1.92
Prior anticoagulation	3.83 (CI 95% 0.45–32.39)	3.55
Abnormal liver function	2.21 (CI 95% 0.23–21.05)	2.04
Drug/ alcohol concomitantly	0.94 (CI 95% 0.18–4.93)	0.94
Bleeding diathesis	1.87 (CI 95% 0.34–10.14)	1.76

Legend. The odds ratio and relative risks of bleeding risk factors have been reported. As mentioned above, the prior anticoagulation carries the highest relative risk between 3 and 4.

#### Multivariate logistic regression

To determine the effect of different risk factors on the bleeding following TDA, we performed a multivariate logistic regression. Only two of 15 independent variables did reach the significance. The female sex (odds ratio .075 and a coefficient − 2.58) and anemia (odds ratio .022 and a coefficient − 3.80) showed a *p*-value lower than 0.05. As the coefficient of these variables is negative, they negatively correlate the risk of bleeding secondary to TDA. The HAS-BLED score (*p* = 0.97) and the modified HAS-BLED score (*p* = 0.13) did not reach the statistical significance in the multivariate regression model.

## Discussion

Anticoagulants are among the most common medications prescribed in clinical practice. Although they are given in order to prevent or treat life-threatening thromboembolic events such as stroke or pulmonary embolism, they could be dangerous if prescribed inappropriately [21]. Owing to the wider therapeutic window and the lower dietary and drug interactions of the newer ACs, which are recently introduced to the pharmaceutical marketing, their use in clinical practice have been surpassed that of vitamin-K-antagonists or heparin. However, lack of specific blood tests with wide availability for monitoring the blood level and therapeutic effects of the newer ACs increases the risk of drug bioaccumulation and, subsequently, the risk of bleeding complications [19]. Owing to being critically harmful in the case of medication errors, the AC- related errors have been largely studied. The adverse drug events commonly occur in clinical practice, with a frequency of 6.5 pro 100 admissions [22]. It has been shown that ACs-related medication errors comprise a significant proportion of medication errors in in-patient setting (7.2–8.3%) and the subsequent adverse events could be prevented in about 70% of cases [3, 22]. Pizza et al. retrospectively analyzed the AC-associated adverse drug events reported to the drug safety system at their tertiary hospital and showed that AC-associated medication errors comprise about half of the adverse drug events owing to AC prescription [3]. In addition, these errors have been significantly correlated with the 30-days mortality rate and higher hospitalization costs [3, 23]. Despite similarities of different reports in showing the high frequency of AC-associated medication errors, there are discrepancies regarding involved AKs, the proportion of error types, and the time of medication process in which errors occur. It seems that the following parameters play a role in the above-mentioned discrepancies: time of study according to the first appearance of the newer AK agents, pharmaceutical market varieties among countries in which the studies have been done, and the difference in study population [2, 24].

Although the therapeutic duplication of ACs (TDA) has been written in medication safety alerts of national and international healthcare authorities [25, 26], there is not any study investigating the frequency and the rate of bleeding complications secondary to TDA. From a total of 1663 patients who have received at least two ACs during a 1-year period, 121 TDA-events have occurred. Out of them, 14 patients have been excluded due to the refusal of participation. The TDA-events have been mainly occurred, in about 88.7% of cases, due to the concomitant prescription of DOACs and LMWHs in prophylactic doses. Consistently, Andreica I. et al. reported a few similar cases in which a new LMWH has been prescribed while the previous DOAC prophylaxis was not discontinued [7]. Among 105 patients treated with heparins, either LMWH or unfractionated heparin, 85 patients received the heparin in prophylactic dose (8 patients with low-dose and other with a prophylactic dose higher than 3400 U/d) and 20 patients in therapeutic dose. Leonardi MJ, et al. in a systematic review of 33 randomized control trial evaluating the bleeding complications of patients under pharmacologic deep vein thrombosis (DVT) prophylaxis showed that patients receiving a high prophylactic dose of LMWH carry a risk of 5.8, 1 and 0.3% for hematuria, gastrointestinal (GI) bleeding and retroperitoneal bleeding, respectively. In comparison, the patients receiving a low dose of LMWH develop in < 0.6% of cases the above-mentioned bleeding complications [27].

We found that the risk for TDA was higher for Rivaroxaban and apixaban compared to other DOACs. Although we do not know the specific reason for this difference, these drugs are routinely prescribed for patients with AF, which may also have a higher likelihood for receiving other anticoagulants. Thus, it is rather the patient population and underlying indication that puts them at risk than the drugs itself.

Although different clinical trials have already investigated the bleeding complications due to each AC class and compared them together, there is not any study reporting the bleeding rate following the concomitant prescription of two different classes i.e. the TDA [9, 11–14]. It has been shown that DOACs, although are not superior to the VKA in the prevention of stroke and systemic embolism, are associated with a significantly lower rate of intracranial bleeding [28–30].

According to the ROCKET-AF study, clinically relevant bleeding occurs in about 14.9% of atrial fibrillation (AF) patients (per year) under rivaroxaban therapy [31]. The ARISTOTLE study assessed the risk of major bleeding complications in AF patients receiving apixaban as prophylaxis for stroke. The study reported a bleeding rate of 0.33, 0.13, 0.68, and 0.01% for intracranial hemorrhage, hematuria, GI bleeding, and retroperitoneal bleeding, respectively [14]. Notably, about 85% of TDA patients received either apixaban or rivaroxaban, mostly as prophylaxis for AF.

Herein, we showed that TDA leads in about 7.5% of cases to Hb-relevant bleeding, defined as a drop more than 10 g/L in hemoglobin value within 24 h, in the first 3 days following the medication error. Pre-existing anemia has been attributed to an increased risk of bleeding secondary to AC therapy [31, 32]. From a clinical standpoint, a bleeding event in anemic patients, especially those with ischemic heart disease, would be more devastating. In our study, 63.5% of patients had anemia and 20.5% suffered from ischemic heart disease (IHD) before a TDA occurred. Furthermore, anemia and IHD were documented in 3 patients with TDA-associated bleeding. It has been demonstrated that AC therapy in patients with deep vein thrombosis leads more frequently to bleeding complications compared to those with atrial fibrillation [19, 20]. It has been attributed to the concomitant polymorbidity and polypharmacy of patients with venous thromboembolic disorders [33, 34]. Among patients with TDA, more than 70% have been subjected to AC-therapy due to atrial fibrillation. Therefore, in a population with a higher proportion of patients having been anticoagulated due to venous thromboembolism (VTE) rather than AF, TDA may lead more frequently to bleeding events compared with the present study.

To assess the risk of bleeding prior to AC therapy, different risk stratification tools have been proposed. Among them, HAS-BLED score, HEMORR2HAGES, and ATRIA are most commonly used in daily clinical practice. Despite having a similar accuracy, the HAS-BLED score has the best predictive performance [35, 36]. These scores have been mainly validated for VKA therapy in patients with atrial fibrillation and, therefore, modified HAS-BLED scores have been proposed to assess the risk of bleeding in other circumstances such as anticoagulation with DOACs [18, 37]. Except for one patient who has been found to concomitantly receive VKA, LMWH, and heparin and one other who received the combination of LMWH and heparin, all other TDA patients have been anticoagulated by DOACs. In addition, 66.3% of TDA patients have been received anticoagulants prior to admission. Prior anticoagulation, though, increases the bleeding risk, has not been considered in the above-mentioned risk stratification tools. Therefore, we measured in addition to the currently used HAS-BLED score, a modified score considering the prior anticoagulant therapy similar to the other medications such as NSAIDs and thrombocyte-aggregation inhibitors, as well.

Interestingly, TDA patients have a mean HAS-BLED score of more than 2 and a mean modified score of more than 3, indicating that the affected population carries a high bleeding risk before TDA-event takes place. In addition to the components of the HAS-BLED score, other clinical parameters predisposing the AK-receiving patients to bleed such as the history of congestive heart failure, cerebrovascular disease, and anemia have also been taken into account. Tamaya et al. in a case-control study of 3252 patients with atrial fibrillation under rivaroxaban-therapy showed that the mentioned parameters, which have been ignored by HAS-BLED score, are the strongest predictive factors for bleeding complications [18]. Consistently, more than 20% of patients with TDA had a positive history of stroke or TIA. In addition, about 18.6% of TDA patients were died at the time of this study i.e. within a maximum of 2 years after discharge from hospital. Altogether, the above-mentioned findings indicate that the involved population carries a high risk of bleeding.

### Case study

Herein, we discuss two cases with TDA-related bleeding.

1. An 80 years-old hypertensive patient presented with frequent episodes of syncope to the emergency department. The clinical examination revealed a hematoma in the right flank and also multiple ecchymosis in different locations. The falling episodes have been attributed to the side effects of Ropinirole, a dopamine agonist prescribed due to Parkinson’s disease. Furthermore, atrial fibrillation has been detected at the time of admission and, therefore, anticoagulation with apixaban (5 mg daily) has begun. As admitted, the patient received also dalteparin in prophylactic dose (5000 IU daily). Just after 1 day of combinative anticoagulation with apixaban and dalteparin the hemoglobin value dropped from 140 g/L to the 117 g/L. The occurrence of gastrointestinal bleeding presenting with melena has been documented during admission. One day later the TDA has been detected and stopped.
2. A 98 years-old hypertensive patient under long-term anticoagulation with rivaroxaban (20 mg daily) due to atrial fibrillation, presented with bilateral subarachnoidal hemorrhage following falling. The patient has been admitted in neurosurgical ward and her medical situation improved without surgical intervention. She received clopidogrel (75 mg daily) following percutaneous transluminal angioplasty, since 1 month ago. Both medications have been stopped at admission. Instead, she received dalteparin in prophylactic dose (2500 IE daily), which has been continued until 1 day before discharge. Clopidogrel and rivaroxaban have been re- started from 4 and 3 days before discharge, respectively. Thus, the patient received for 2 days the combination of Clopidogrel, rivaroxaban and dalteparin, which resulted in Hb-drop from 86 g/L to the 66 g/L (= the Hb-value at first day of TDA) within 48 h. The Hb-value after the second day of TDA is not available. The patient was still disoriented at discharge and has been referred to the nursing house. Finally, she died 3 days after discharge. However, the reason of death is not documented, whether it occurred due to the deterioration of the pre-existing intracranial hemorrhage or/ and new bleeding events or not.

## Conclusion

TDA is a known medication error of anticoagulants with unknown frequency and clinical importance. We retrospectively analyzed the frequency and complications of TDA in all patients admitted to our 450-bed referral hospital in 1 year. TDA occurred in 0.8% of patients who were under anticoagulation and in 6.7% of patients who received DOACs. TDA led in about 7.4% of cases to hemoglobin-relevant bleeding. The old patients with significant comorbidities and a high HAS-BLED score were mainly affected. The female gender and presence of anemia independently predicted the occurrence of bleeding following TDA. Moreover, we introduced a new risk stratification tool by modifying the HAS-BLED score to assess the risk of bleeding in patients who are already on anticoagulants. Although the significance of the proposed tool is the matter of further investigations with larger sample sizes, our analyses suggest the consideration of prior anticoagulation as a parameter in addition to the HAS-BLED score lead to the prediction of the bleeding risk in TDA patients at a higher level of significance.

## Data Availability

The datasets used and/or analysed during the current study are available from the corresponding author on reasonable request.

## Abbreviations

TDA: therapeutic duplication of anticoagulants
AC: anticoagulants,
LMWH: low-molecular weight heparins
DOAC: direct oral anticoagulants
VKA: vitamin-K antagonists
DVT: deep vein thrombosis
IHD: ischemic heart disease
VTE: venous thromboembolism
AF: atrial fibrillation

## References

[1] Institute for Safe Medication Practices Canada. ISMP List of High-Alert Medications in Acute Care Settings. Inst Safe Medicat Pract. 2014.

[2] Dreijer AR, Diepstraten J, Bukkems VE, Mol PGM, Leebeek FWG, Kruip MJHA, et al. Anticoagulant medication errors in hospitals and primary care: a cross-sectional study. Int J Qual Heal Care. 2019;31(5):346–52. 10.1093/intqhc/mzy177 30165484

[3] Piazza G, Nguyen TN, Cios D, Labreche M, Hohlfelder B, Fanikos J, et al. Anticoagulation-associated adverse drug events. Am J Med. 2011;124(12):1136–42. 10.1016/j.amjmed.2011.06.009 PMC3224344 22114827

[4] Barr D, Epps QJ. Direct oral anticoagulants: a review of common medication errors. J Thromb Thrombolysis. 2019;47(1):146–54. 10.1007/s11239-018-1752-9 30298305

[5] Henriksen JN, Nielsen LP, Hellebek A, Poulsen BK. Medication errors involving anticoagulants: data from the Danish patient safety database. Pharmacol Res Perspect. 2017;5(3):e00307. 10.1002/prp2.307 PMC5464338 28603628

[6] Tran E, Duckett A, Fisher S, Bohm N. Appropriateness of direct oral anticoagulant dosing for venous thromboembolism treatment. J Thromb Thrombolysis. 2017;43(4):505–13. 10.1007/s11239-017-1487-z 28271315

[7] Andreica, Ivyruth; Grissinger M. Oral Anticoagulants: A Review of Common Errors and Risk Reduction Strategies. Pennsylvania Patient Saf Auth. 2015;12(2):54–61.

[8] Grissinger M, Gaunt MJ, Rich DS. Avoiding medication errors: reducing harm in residents using oral anticoagulants. Consult Pharm. 2016;31(6):294–303. 10.4140/TCP.n.2016.294 27250070

[9] Desai RJ, Williams CE, Greene SB, Pierson S, Hansen RA. Anticoagulant medication errors in nursing homes: characteristics, causes, outcomes, and association with patient harm. J Healthc Risk Manag. 2013;33(1):33–43. 10.1002/jhrm.21116 23861122

[10] Uppuluri EM, McComb MN, Shapiro NL. Implementation of a direct Oral anticoagulation screening Service at a Large Academic Medical Center Provided by a pharmacist-managed Antithrombosis clinic as a method to expand antithrombotic stewardship efforts. J Pharm Pract. 2020;33(3):271–5. 10.1177/0897190018799200 30213217

[11] Eriksson BI, Borris LC, Friedman RJ, Haas S, Huisman M V., Kakkar AK, et al. Rivaroxaban versus enoxaparin for thromboprophylaxis after hip arthroplasty. N Engl J Med. 2008;358:2765–75. 10.1056/NEJMoa0800374 18579811

[12] Landman GW, Gans ROB. Oral rivaroxaban for symptomatic venous thromboembolism. New England Journal of Medicine. 2011;364(12):1178. 10.1056/NEJMc1100734 21428778

[13] Agnelli G, Buller HR, Cohen A, Curto M, Gallus AS, Johnson M, et al. Oral apixaban for the treatment of acute venous thromboembolism. N Engl J Med. 2013;369(9):799–808. 10.1056/NEJMoa1302507 23808982

[14] Granger CB, Alexander JH, McMurray JJV, Lopes RD, Hylek EM, Hanna M, et al. Apixaban versus warfarin in patients with atrial fibrillation. N Engl J Med. 2011;365:981–92. 10.1056/NEJMoa1107039 21870978

[15] Narum S, Solhaug V, Myhr K, Johansen PW, Brørs O, Kringen MK. Warfarin-associated bleeding events and concomitant use of potentially interacting medicines reported to the Norwegian spontaneous reporting system. Br J Clin Pharmacol. 2011;71(2):254–62. 10.1111/j.1365-2125.2010.03827.x PMC3040547 21219407

[16] Teklay G, Shiferaw N, Legesse B, Bekele ML. Drug-drug interactions and risk of bleeding among inpatients on warfarin therapy: a prospective observational study. Thromb J. 2014;12:20. 10.1186/1477-9560-12-20 PMC4171718 25249791

[17] Fusco JA, Paulus EJ, Shubat AR, Miah S. Warfarin and Rivaroxaban Duplication: A Case Report and Medication Error Analysis. Drug Saf - Case Reports. 2015;2(1):5. 10.1007/s40800-015-0007-3 PMC5005698 27747717

[18] Tamayo SG, Simeone JC, Nordstrom BL, Patel MR, Yuan Z, Sicignano NM, et al. Risk Factors for Major Bleeding in Rivaroxaban Users With Atrial Fibrillation. Journal of the American College of Cardiology. 2016;68(10):1144–6. 10.1016/j.jacc.2016.06.028 27585515

[19] Crowther MA, Warkentin TE. Bleeding risk and the management of bleeding complications in patients undergoing anticoagulant therapy: focus on new anticoagulant agents. Blood. 2008;111(10):4871–9. 10.1182/blood-2007-10-120543 18309033

[20] Shoeb M, Fang MC. Assessing bleeding risk in patients taking anticoagulants. In: J Thrombosis Thrombolysis. 2013;35(3):312–9. 10.1007/s11239-013-0899-7 PMC3888359 23479259

[21] Landefeld CS, Beyth RJ. Anticoagulant-related bleeding: clinical epidemiology, prediction, and prevention. Am J Med. 1993;95(3):315–28. 10.1016/0002-9343(93)90285-w 8368229

[22] Poon EG, Cina JL, Churchill W, Patel N, Featherstone E, Rothschild JM, et al. Medication dispensing errors and potential adverse drug events before and after implementing bar code technology in the pharmacy. Ann Intern Med. 2006;145(6):426–34. 10.7326/0003-4819-145-6-200609190-00006 16983130

[23] Bates DW, Spell N, Cullen DJ, Burdick E, Laird N, Petersen LA, et al. The costs of adverse drug events in hospitalized patients. J Am Med Assoc. 1997;277(17):1351. 9002493

[24] Budnitz DS, Lovegrove MC, Shehab N, Richards CL. Emergency hospitalizations for adverse drug events in older Americans. N Engl J Med. 2011;365:2002–12. 10.1056/NEJMsa1103053 22111719

[25] Quick-Alert 41, Stiftung für Patientensicherheit, Schweiz.

[26] Commission TJ. National Patient Safety Goals Effective January 1 , 2015. Hosp Natl Patient Saf Goals. 2015.

[27] Leonardi MJ, McGory ML, Ko CY. The rate of bleeding complications after pharmacologic deep venous thrombosis prophylaxis: a systematic review of 33 randomized controlled trials. Arch Surg. 2006;141(8):790–7. 10.1001/archsurg.141.8.790 16924087

[28] Ruff CT, Giugliano RP, Braunwald E, Hoffman EB, Deenadayalu N, Ezekowitz MD, et al. Comparison of the efficacy and safety of new oral anticoagulants with warfarin in patients with atrial fibrillation: a meta-analysis of randomised trials. Lancet. 2014;383(9921):955–62. 10.1016/S0140-6736(13)62343-0 24315724

[29] Wang YP, Kehar R, Iansavitchene A, Lazo-Langner A. Bleeding risk in non-Valvular atrial fibrillation patients receiving direct Oral anticoagulants and warfarin: a systematic review and meta-analysis of observational studies. Blood. 2019;134(Supplement_1):3672. 10.1055/s-0040-1714918 PMC7358046 32676543

[30] Lobraico-Fernandez J, Baksh S, Nemec E. Elderly bleeding risk of direct Oral anticoagulants in Nonvalvular atrial fibrillation: a systematic review and meta-analysis of cohort studies. Drugs in R and D. 2019;19(3):235–45. 10.1007/s40268-019-0275-y PMC6738514 31127504

[31] Patel MR, Mahaffey KW, Garg J, Pan G, Singer DE, Hacke W, et al. Rivaroxaban versus warfarin in nonvalvular atrial fibrillation. N Engl J Med. 2011;365:883–91. 10.1056/NEJMoa1009638 21830957

[32] Beyth RJ, Quinn LM, Landefeld CS. Prospective evaluation of an index for predicting the risk of major bleeding in outpatients treated with warfarin. Am J Med. 1998;105(2):91–9. 10.1016/s0002-9343(98)00198-3 9727814

[33] Levine MN, Raskob G, Landefeld S, Kearon C. Hemorrhagic complications of anticoagulant treatment. Chest. 2001;119(1 Suppl):108S–121S. 10.1378/chest.119.1_suppl.108s 11157645

[34] Lassen MR, Gallus A, Raskob GE, Pineo G, Chen D, Ramirez LM. Apixaban versus enoxaparin for thromboprophylaxis after hip replacement. N Engl J Med. 2010;363(26):2487–98. 10.1056/NEJMoa1006885 21175312

[35] Hellenbart EL, Faulkenberg KD, Finks SW. Evaluation of bleeding in patients receiving direct oral anticoagulants. Vasc Health Risk Manag. 2017;13:325–42. 10.2147/VHRM.S121661 PMC5574591 28860793

[36] Lip GYH, Nieuwlaat R, Pisters R, Lane DA, Crijns HJGM, Andresen D, et al. Refining clinical risk stratification for predicting stroke and thromboembolism in atrial fibrillation using a novel risk factor-based approach: the euro heart survey on atrial fibrillation. Chest. 2010;137(2):263–72. 10.1378/chest.09-1584 19762550

[37] Tsu LV, Berry A, Wald E, Ehrlich C. Modified HAS-BLED score and risk of major bleeding in patients receiving dabigatran and rivaroxaban: a retrospective, case-control study. Consult Pharm. 2015;30(7):395–402. 10.4140/TCP.n.2015.395 26173191

